# Herbivory facilitates growth of a key reef‐building Caribbean coral

**DOI:** 10.1002/ece3.3620

**Published:** 2017-11-22

**Authors:** Adam Suchley, Lorenzo Alvarez‐Filip

**Affiliations:** ^1^ Posgrado en Ciencias del Mar y Limnología Universidad Nacional Autónoma de México Mexico City Mexico; ^2^ Biodiversity and Reef Conservation Laboratory Unidad Académica de Sistemas Arrecifales Instituto de Ciencias del Mar y Limnología Universidad Nacional Autónoma de México Puerto Morelos Mexico

**Keywords:** calcification, coral reefs, coral–macroalgal competition, *Dictyota*, herbivory, *Orbicella*

## Abstract

The decline of reef‐building corals in conjunction with shifts to short‐lived opportunistic species has prompted concerns that Caribbean reef framework‐building capacity has substantially diminished. Restoring herbivore populations may be a potential driver of coral recovery; however, the impact of herbivores on coral calcification has been little studied. We performed an exclusion experiment to evaluate the impact of herbivory on *Orbicella faveolata* coral growth over 14 months. The experiment consisted of three treatments: full exclusion cages; half cage procedural controls; and uncaged control plates, each with small *O. faveolata* colonies. We found that herbivorous fish exclusion had a substantial impact on both macroalgal cover and coral growth. Fleshy macroalgae reached 50% cover within some exclusion cages, but were almost absent from uncaged control plates. Critically, *O. faveolata* calcification rates were suppressed by almost half within exclusion cages, with monthly coral growth negatively related to overgrowth by fleshy macroalgae. These findings highlight the importance of herbivorous fishes for coral growth and the detrimental impact of macroalgal proliferation in the Caribbean. Policy makers and local managers should consider measures to protect herbivorous fishes and reduce macroalgal proliferation to enable coral communities to continue to grow and function.

## 1 INTRODUCTION

On coral reefs, scleractinian corals perform an extensive ecosystem engineering role through the calcification of structurally complex physical structures (Graham & Nash, 2013; Jones, Lawton, & Shachak, 1994). Corals provide habitat for diverse fauna including herbivorous fishes, which are key organisms as they control the abundance and community structure of macroalgae which compete with corals (Hughes, 1994; Jackson, Donovan, Cramer, & Lam, 2014; Paddack et al., 2009). Although the impact of herbivory on algae has been well studied, facilitation of coral growth and calcification is poorly understood (Burkepile & Hay, 2006; Fong & Paul, 2011; McCook, Jompa, & Diaz‐Pulido, 2001). Explaining the role of herbivores in facilitating coral ecosystem engineering is of fundamental ecological interest and can inform reef conservation strategies seeking to maintain ecosystem function.

In recent decades, coral reefs have experienced major declines in live coral cover resulting from climate change, coral diseases, herbivore decline, and coastal development (Aronson & Precht, 2001; Hoegh‐Guldberg et al., 2007; Jackson et al., 2014; Mora, 2008). Furthermore, Caribbean reefs have undergone shifts in coral community composition to dominance by short‐lived and slower growing species that contribute less to community calcification (Alvarez‐Filip, Carricart‐Ganivet, Horta‐Puga, & Iglesias‐Prieto, 2013; Bruckner & Bruckner, 2006; Edmunds & Elahi, 2007). Coral declines are often accompanied by rapid increases in macroalgae that impair the capacity of coral communities to recover (Birrell, Mccook, Willis, & Diaz‐Pulido, 2008; Done, 1992; Hughes, Reed, & Boyle, 1987). Such phase shifts have been particularly evident in the Caribbean where herbivory has been substantially reduced from historical baselines (Jackson et al., 2001; Paddack et al., 2009). A previously key Caribbean herbivore, the sea urchin *Diadema antillarum*, experienced a mass mortality event in 1983/4 and has recovered at few sites across the region (Idjadi, Haring, & Precht, 2010; Jackson et al., 2001; Lessios, 2016). Parrotfishes, surgeonfishes, and sea urchins are considered the dominant herbivores on today's Caribbean reefs (Hughes, Graham, Jackson, Mumby, & Steneck, 2010; Kuempel & Altieri, 2017; Sangil & Guzman, 2016); however, overfishing has considerably diminished reef fish populations (Jackson et al., 2001; Paddack et al., 2009).

Diminished grazing capacity on Caribbean reefs has prompted extensive evaluation of the role of herbivores in algal control by both observational and experimental studies. Observational studies cover a broad range of spatial and temporal scales and provide evidence for (Jackson et al., 2014; Newman, Paredes, Sala, & Jackson, 2006) and against (Cox, Valdivia, McField, & Bruno, 2017; Russ, Sarah‐Lee, Rizzari, & Alcala, 2015; Suchley, McField, & Alvarez‐Filip, 2016) the ability of grazers to control benthic algae. Herbivore exclusion has become a standard technique to experimentally assess the ability of both fishes and invertebrates to graze algae and has been adopted by many studies. These small‐scale experimental studies generally involve the exclusion of herbivores via caging of experimental plots or artificial substrate units over time periods ranging from days to years. In contrast to observational studies, experimental studies consistently tend to report a significant effect of herbivores on turf and macroalgal proliferation, with other factors such as nutrient levels and seasonality playing secondary roles (Burkepile & Hay, 2006, 2009; Ferrari, Gonzalez‐Rivero, Ortiz, & Mumby, 2012; Sotka & Hay, 2009).

Phase shifts from coral to algal dominance experienced by many Caribbean reefs have underlined the importance of the interaction between algae and scleractinian corals (Arias‐González et al., 2017; Done, 1992; Hughes, 1989). However, despite the importance of the herbivore‐algal‐coral interaction, experimental evidence of the impact of herbivory on coral calcification is limited. In the Caribbean, *Acropora* spp. were major reef framework builders historically, but subsequent to a wide‐spread epizootic event in the 1970/80s populations remain severely diminished (Alvarez‐Filip, Dulvy, Côte, Watkinson, & Gill, 2011; Aronson & Precht, 2001; Gladfelter, 1982). Following the loss of *Acropora*, species of the genus *Orbicella* are the major framework builders on many of today's Caribbean reefs (McClanahan & Muthiga, 1998; Perry et al., 2012; Porto‐Hannes et al., 2015). Although the effect of herbivory on *Orbicella* spp. has been considered (Foster, Box, & Mumby, 2008; Lirman, 2001; Vermeij et al., 2010), the impact of herbivory on coral calcification has seldom been assessed (Vu et al., 2009). To address this knowledge gap, we performed a 14‐month herbivore exclusion experiment to determine the effect of fish herbivory on *Orbicella faveolata* calcification. Our hypothesis is that herbivorous fishes restrict algal growth and that algal interaction has a negative impact on coral growth and calcification.

## 2 MATERIALS AND METHODS

This study was conducted over a 14‐month period from August 2015 to October 2016 in the “La Bocana” back reef located in the Parque Nacional Arrecife de Puerto Morelos, Mexico (20°52′26″N, 86°51′5″W). We reviewed national park monitoring data from the previous 3 years and preliminary surveys were performed at three sites within the park to evaluate the abundance of sea urchins and the biomass of fishes of the Scaridae and Acanthuridae families. Sea urchins were not observed at any site. Consequently, “La Bocana” back reef patch was selected as it displayed among the highest herbivorous fish biomass (average of 3,600 g/100 m^2^ from national park monitoring and preliminary survey data). La Bocana reef patch is at a depth of 4 m and is characterized by large *Orbicella faveolata* colonies, *Agaricia* spp., dead *Acropora palmata* framework, gorgonians, and an algal community dominated by brown macroalgae, turf algae, green calcareous *Halimeda* spp., and red calcareous *Amphiroa* spp. Preliminary surveys and national park monitoring revealed reef patch coral cover of approximately 17% and fleshy macroalgal cover of 17%.

To test the effect of fish herbivory on coral growth, fishes were excluded from experimental units containing small *O. faveolata* colonies. Half‐ellipsoid *O. faveolata* colonies (approximately 5 cm maximum diameter) were collected prior to 2011 from the same site by removing fragments from large colonies and were left in the reef lagoon for later use. The experimental design constituted three treatments with replicate numbers limited by coral colony availability: (1) full cages excluded herbivorous fishes (*n* = 6); (2) half cages with caging at sides but no top panel acted as procedural controls testing for caging effects by allowing fish access (*n* = 6); (3) plates with no caging acted as full controls (*n* = 6). Cages were built upon 36‐cm‐square concrete plates, with 30‐cm vertical steel reinforcing bars and thin 3 cm × 2 cm polyethylene mesh (Fig. S1). Concrete is commonly used as an artificial substrate in herbivore exclusion experiments (Burkepile & Hay, 2009, 2010; Rasher et al., 2012). In late August 2015, coral colonies were retrieved and wet‐weighed in the laboratory. Cages and plates were randomly located in the reef patch over an area of approximately 400 m^2^, with a minimum separation of 2 m, and coral colonies were affixed to plate centers using underwater epoxy plasticine (Fig. S1). At the end of the observation period, coral colonies were removed from plates and reweighed in order to determine net calcification rates (in g cm^−2^ year^−1^, based on average colony area over the observation period). Underwater Hobo data loggers tracked in situ water temperature at cage depth during the experiment, and the impact of caging on light levels was evaluated using three underwater Hobo data loggers in a cage, a half cage and on a control plate during a sunny day at the end of June 2016. Light levels were converted from Lux to μmol quanta m^−2^ s^−1^ by applying a conversion factor of 54 (Sager & McFarlane, 1997). Light levels were compared between treatments using a Friedman test for repeated measures due to non‐normality.

Cages were monitored at the beginning of every month for 14 months and the mesh cleaned of algae and other fouling organisms with brushes every 1 to 2 weeks during this period. Every month top‐down photographs of the plates were taken in order to assess algal cover. The software photoQuad v1.4 was used to determine percentage cover by area of a 30‐cm quadrat contained within cages and on control plates by tracing algal mini‐patch perimeters (Trygonis & Sini, 2012). Plate cover components were classified as short (<0.5 cm) or long (≥0.5 cm) filamentous turf algae; brown, green, or red fleshy macroalgae (to species or genus level); crustose coralline algae (CCA); calcareous algae; cyanobacteria; or gorgonian.

To identify which herbivorous fishes were browsing plate algae, in situ feeding was recorded by static GoPro video cameras. Observations were performed over 1‐hr intervals for a total of 3 hr for full cages, 3 hr for half cages, and 8 hr for uncaged control plates, between May and August 2016 between 11 a.m. and 4 p.m. For each 1‐hr interval, feeding fish bite counts were recorded by species, total length (size categories estimated based on plate size), and bite target. Individual bites were defined as those involving a rapid movement of the head toward and away from the plate (Bellwood & Fulton, 2008). Fish biomass was determined using standard allometric length‐weight conversions. Bite counts were subsequently converted to estimates of herbivory pressure by standardizing (multiplying) by herbivore biomass following Bellwood, Hughes, and Hoey (2006). Herbivory pressure was compared between treatments based on the first three hours of concurrent observation to standardize for temporal variation in fish activity. Herbivore identity evaluation was performed for control plates only (based on 8 hr of observation) due to low levels of consumption within exclusion cages.

Every month close‐up photographs of coral colonies (with a ruler for scale) were taken in order to evaluate coral planar area (substrate area occupied by colonies), perform polyp counts using the software ImageJ, and count occurrences of algal overgrowth (Rasband, 2014). Photos were taken with consistent orientation to minimize variation in coral area and polyp counts due to camera angle. Corals were overgrown by *Dictyota* spp. and/or turf algae/sediment mix (also known as the “epilithic algal matrix”). Each month overgrowth was assessed and corals classified as overgrown if any partial overgrowth was evident. As colony perimeters were obscured by algal overgrowth it was not possible to quantify interactions by coral area affected. Incidence of disease, coral bleaching, mortality, and algal overgrowth was recorded.

**Figure 1:**
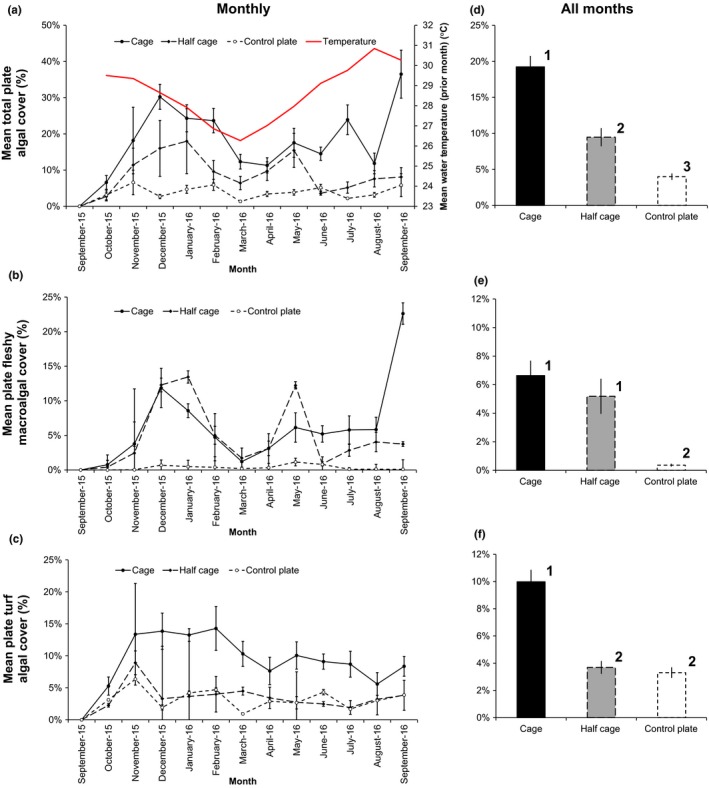
Total algal, fleshy macroalgal and turf algal plate mean cover and temporal trends. (a–c) Mean (±*SEM*) monthly total plate algal cover (a), fleshy macroalgal cover (b), and turf algal cover (c) for full exclusion cages (*n *=* *6), half cage controls (*n* = 6), and uncaged control plates (*n* = 6). Algal cover monitoring was performed at the beginning of each month. Mean water temperature for the prior month measured by Hobo data loggers is also shown. (d–f) Mean (±*SEM*) total plate algal cover (d), fleshy macroalgal cover (e), and turf algal cover (f) over the observation period for full cages (*n* = 6), half cages (*n* = 6), and uncaged control plates (*n* = 6). Numbers represent significant differences indicated by mixed model Tukey post hoc testing (Table 2). Note the difference in scale between panels (a) and (d) and other panels

Mixed effect models were fitted to evaluate the influence of experimental treatment and water temperature on monthly total, fleshy macroalgal, and turf algal cover, with month and replicate included as random effects. Algal cover was evaluated at the beginning of each month, and consequently, the mean water temperature for the prior month was utilized. The algal cover time series were truncated to begin in December 2015 to account for initial algal growth after cage installation (Figure 1). Linear model assumptions were assessed using residual diagnostic plots. All algal percent cover variables were arcsin‐sqrt transformed due to non‐normality of residuals. Interaction terms between treatment and temperature were tested and subsequently removed if found not to be significant. ANOVAs were applied to the fitted models to determine variable‐level significance and Tukey post hoc testing was performed to compare effects between treatments.

Comparison of coral calcification between treatments was performed using Welch's *t* tests or non‐parametric Mann–Whitney *U* tests, based on an assessment of normality using Shapiro–Wilk tests. Initial coral colony planar area, polyp count, and mass were compared between treatments using ANOVAs, with assumptions checked using diagnostic plots. To determine the drivers of coral growth over time, coral polyp count was preferred to coral planar area as it was observed to be a more consistent measure of individual colony growth. Mixed effect models were fitted to evaluate the drivers of month‐on‐month coral polyp count change. Experimental treatment, water temperature, plate fleshy macroalgal and turf algal cover, fleshy macroalgal, and turf algal overgrowth of coral colonies were included as fixed effects, with month and replicate included as random effects. One‐month lagged versions of algal cover and overgrowth were also included as candidate predictor variables. The time series was truncated to begin in November 2015 as October 2015 was used as the baseline for polyp counts. Linear model assumptions were assessed using residual diagnostic plots. Multicollinearity among predictor variables was tested using variance inflation factors (VIFs), but no evidence of multicollinearity was observed (Graham, 2003). Least significant predictors were sequentially removed and models compared pairwise with partial *F* tests of significant differences in error sum‐of‐squares. The more parsimonious model was preferred until the partial *F* test revealed a significant difference and the prior model retained as the minimum adequate model. All statistical analyses were performed using R version 3.3.2 (R Core Team 2016) using packages including *lme4* and *nlme* for mixed effect modeling, *car* for VIF assessment, *multcomp* for Tukey post hoc testing, and *r2glmm* for mixed effect model partial R‐squared.

## 3 RESULTS

### 3.1 Effect of herbivore exclusion on macroalgal cover

Experimental units were installed in late August 2015 and were subsequently rapidly colonized by turf algae and macroalgae (Figure 1). Averaging across all monthly observation periods and plates, mean plate turf algal cover was 5.7%; fleshy macroalgae, 4.1%; cyanobacteria, 1.0%; CCA, 0.1%; and calcareous algae, 0.01%. Cyanobacteria were subject to short‐term blooms such as in July 2016 which resulted in sudden increases in cover to over 20% in some cages. Fleshy macroalgal cover was dominated by *Dictyota* spp. (82% mean cover relative to total fleshy macroalgae) and showed substantial variation over time reaching above 50% total cover within some cages (Figure 1b), while turf algal cover appeared more stable over time (Figure 1c).

Total algal cover, fleshy macroalgal cover, and turf algal cover varied significantly by experimental treatment (Figure 1; Table 1). Algal cover was consistently significantly higher in full exclusion cages than on uncaged control plates (Figure 1d–f; Table 2). On average, macroalgal cover was 1731% relatively higher and turf algal cover was 202% higher in full exclusion cages than on uncaged control plates (Figure 1e,f). While there was no significant difference in fleshy macroalgal cover between full and half cages (Figure 1e; Table 2), turf algal cover was significantly (171%) higher in full cages than half cages (Figure 1f; Table 2). Mean short (<0.5 cm) and long (≥0.5 cm) algal turf cover displayed similar relative levels between treatments (Fig. S2). The interaction of water temperature and cage type had a significant effect on fleshy macroalgal cover (Figure 1b; Table 1).

**Table 1:** Monthly algal cover mixed modeling. Plate monthly total algal cover, fleshy macroalgal cover, and turf algal cover between December 2015 and September 2016 were modeled as a function of treatment (cage type) and water temperature (mean temperature for the prior month), with month and replicate as random effects. Table shows ANOVA model summaries. The time series were truncated to December 2015 to account for initial algal growth after cage installation (Figure 1). Interaction terms were removed from the models if non‐significant for all variable categories. Asterisks denote level of significance (*denotes *p* < .05 and **denotes *p* < .01)

Dependent variable	Predictor	*F*‐value	*df*	*p*
Total algal cover	(Intercept)	342.59	1	<.001**
	Cage type	22.11	2	<.001**
	Temperature	0.37	1	.545
Fleshy macroalgal cover	(Intercept)	48.07	1	<.001**
	Cage type	7.36	2	.006**
	Temperature	2.60	1	.109
	Cage type × temperature	6.06	2	.003**
Turf algal cover	(Intercept)	944.28	1	<.001**
	Cage type	47.65	2	<.001**
	Temperature	2.67	1	.104
	Cage type × temperature	2.64	2	.075

**Table 2:** Effect of experimental treatment on monthly algal cover. Plate monthly total algal cover, fleshy macroalgal cover, and turf algal cover compared between experimental treatments by applying Tukey post hoc tests to fitted mixed models (Table 1). Experimental treatments are full exclusion cages (*n* = 6), half cage controls (*n* = 6), and uncaged control plates (*n* = 6). Asterisks denote level of significance (* denotes *p* < .05 and ** denotes *p* < .01)

Dependent variable	Comparison	*p*
Total algal cover	Cage versus control plate	<.001**
	Cage versus half cage	<.001**
	Half cage versus control plate	.023*
Fleshy macroalgal cover	Cage versus control plate	<.001**
	Cage versus half cage	.479
	Half cage versus control plate	.026*
Turf algal cover	Cage versus control plate	<.001**
	Cage versus half cage	<.001**
	Half cage versus control plate	.831

### 3.2 Herbivorous fish identity

In situ herbivore feeding observations revealed grazing on plates by herbivorous fishes. Uncaged control plates experienced an average of 87 bites/hr, and both full and half cages were subject to 63 bites/hr, with the majority (87%) directed at turf algae. Fishes up to 10 cm total length (TL) were observed to enter full cages through the 3 cm × 2 cm mesh. *Acanthurus* spp. larger than 10 cm TL entered half cages from above, although these did not feed on half cage plate algae. Consequently, less herbivory pressure was observed within full and half cages (64 and 520 bites g hr^−1^ plate^−1^ respectively) compared with control plates (6,874 bites g hr^−1^ plate^−1^; Fig. S3). The majority (62.4%) of turf algal consumption on control plates was performed by *Acanthurus bahianus* surgeonfish, with juvenile *Scarus iseri* parrotfish (25.5%), *A. coeruleus* surgeonfish (9.1%), and juvenile *Sparisoma aurofrenatum* parrotfish (2.9%) carrying out lesser roles. Control plates displayed very low fleshy macroalgal cover and so an equivalent analysis for macroalgae consumers, in addition to feeding electivity estimation (sensu Adam, Kelley, Ruttenberg, & Burkepile, 2015), was not possible.

### 3.3 Effect of herbivore exclusion on coral growth and calcification

**Figure 2:**
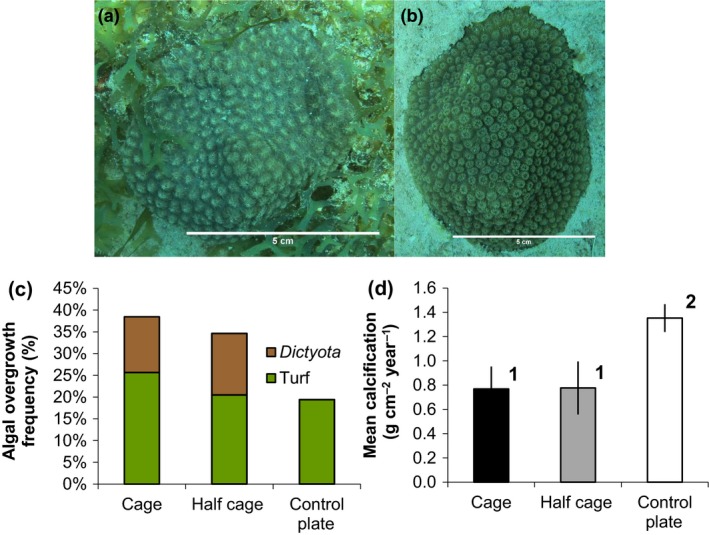
Coral‐algal interactions and *Orbicella faveolata* colony calcification rates. (a) *O. faveolata* colony within a herbivore exclusion cage overgrown by the fleshy macroalgae *Dictyota* spp. (b) *O. faveolata* colony on an uncaged control plate partially covered by the surrounding turf algae/sediment mix along colony perimeter. (c) Percentage of monthly coral observations recording partial algal overgrowth. Algal overgrowth is classified as partial overgrowth by *Dictyota* or turf algae/sediment mix. (d) Mean (±*SEM*) net calcification rates calculated as annualized change in colony mass by average colony planar area over the study period from August 2015 to October 2016 for full exclusion cages (*n* = 6), half cage controls (*n* = 6), and uncaged control plates (*n* = 5, as one colony was lost after 2 months).

Initial *Orbicella faveolata* colony planar area, polyp count, and mass were not significantly different between treatments (ANOVA, coral area, *F*_2,14_ = 0.44, *p* = .65; polyp count, *F*_2,14_ = 0.95, *p* = .41; mass, *F*_2,14_ = 1.05, *p* = .38; Fig. S4). At the end of the observation period, coral calcification was significantly (43%) lower for colonies enclosed by full and half cages than those located on uncaged control plates subject to herbivory (Welch's *t* test, control plate vs. full cage, *t* = 2.68, *df* = 8.1, *p* = .03; control plate vs. half cage, *t* = 2.34, *df* = 7.4, *p* = .05; Figure 2d). Calcification was not significantly different between colonies located in full cages and those in half cages (Welch's *t* test, *t* = 0.03, *df* = 9.8, *p* = .98; Figure 2d).

**Figure 3:**
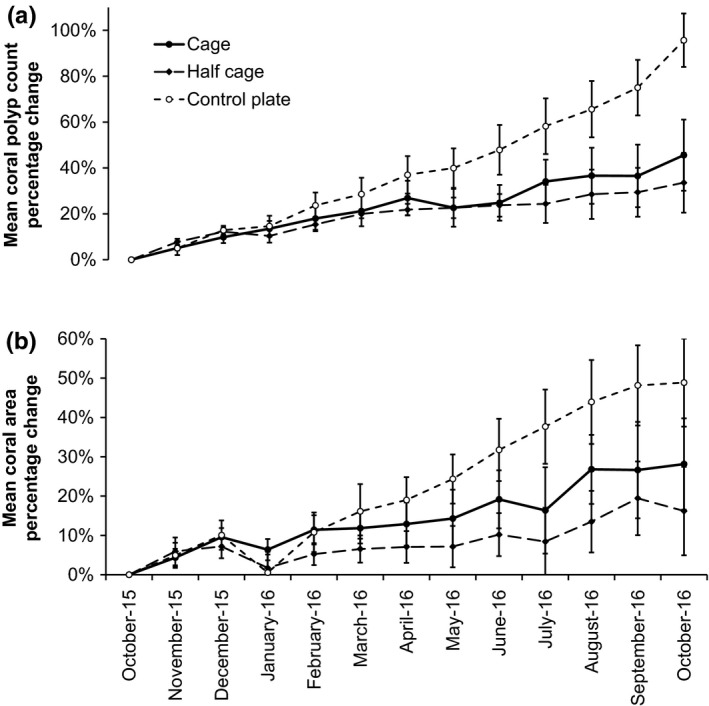
*Orbicella faveolata* colony growth temporal trend. (a) Mean (±*SEM*) coral polyp count percentage change over the study period. (B) Mean (±*SEM*) coral planar area percentage change over the study period. Colony growth was determined at the beginning of each month for full exclusion cages (*n* = 6), half cage controls (*n* = 6), and uncaged control plates (*n* = 5, as one colony was lost after 2 months)

Interactions between *Orbicella faveolata* colonies and surrounding algae were commonly observed (Figure 2). Within cages, coral colonies were often partially overgrown by *Dictyota* spp. and/or the surrounding turf algae/sediment mix (Figure 2a,c). *Dictyota* spp. were the only fleshy macroalgae observed overgrowing corals. All instances of overgrowth by *Dictyota* also involved turf algae/sediment mix to some extent. For uncaged control plates, overgrowth by *Dictyota* was not observed, and only coral colony perimeters were partially covered by the surrounding turf algae/sediment mix (Figure 2b,c). Coral polyp counts and planar area were assessed on a monthly basis and show generally linear increases over time (Figure 3). Cage type and overgrowth by *Dictyota* had a significant effect on monthly polyp count change while other candidate predictor variables (water temperature, plate fleshy macroalgal and turf algal cover, turf algal overgrowth, and one‐month lagged versions of algal cover and overgrowth) were not significant and were removed from the model (Table 3). The interaction of cage type and *Dictyota* overgrowth was tested and found not to be significant. Further interaction term testing was limited by model degrees of freedom.

**Table 3:** Coral colony polyp count change mixed model coefficient estimates. Month‐on‐month coral colony polyp count change between October 2015 and September 2016 was modeled as a function of treatment (cage type), temperature, plate fleshy macroalgal and turf algal cover, fleshy macroalgal (*Dictyota* spp.), and turf algal overgrowth of coral colonies, with month and replicate as random effects. Uncaged control plates were selected as the base level for the cage type variable. Non‐significant predictor variables were removed from the model. The time series was truncated to use October 2015 as a baseline for polyp counts. Asterisks denote level of significance (* denotes *p* < .05 and ** denotes *p* < .01)

Predictor	Estimate	*p*	Partial *R*‐squared
Intercept	16.309	<.001**	
Cage type: half cage	−8.614	.030*	0.093
Cage type: full cage	−7.852	.045*	0.077
*Dictyota* overgrowth	−9.957	.007**	0.062

Over the study period, no coral mortality (except for colony perimeter tissue loss) occurred and few disease symptoms were observed. Coral bleaching in late summer 2016 was limited to two polyps on one *Orbicella faveolata* colony. Caging was observed to have an effect on light intensity (Friedman test, chi‐squared = 722, *df* = 2, *p* < .001). On a sunny day at the end of June 2016, mean daylight incident at uncaged control plates (408.6 μmol quanta m^−2^ s^−1^) was slightly (4.7%) higher than within half cage procedural controls (390.3 μmol quanta m^−2^ s^−1^), which in turn was (10.9%) greater than within full exclusion cages (352.0 μmol quanta m^−2^ s^−1^).

## 4 DISCUSSION

The substantial impact of reduced fish herbivory on algal cover mediates growth rates of the important reef‐building coral, *Orbicella faveolata*. Over our 14‐month study, macroalgal and turf algal cover were greater within herbivore exclusion cages than on uncaged control plates (Figure 1; Table 2). In situ observations revealed negligible herbivory pressure within exclusion cages (Fig. S3). Fleshy macroalgae were almost always absent from uncaged control plates while cover reached over 50% within some herbivore exclusion cages. Coral‐algal interactions were common within cages and caged coral colonies experienced significantly lower growth than those on control plates (Figures 2 and 3; Table 3). Net annual calcification rates on uncaged control plates were comparable with benchmark values, while in both full and half cages calcification was reduced by over 40% (Figure 2; Perry et al., 2012).

Our findings suggest that herbivorous fishes are critical to the maintenance of coral calcification rates. *Orbicella* spp. are major framework builders on Caribbean reefs, and *O. faveolata* exhibit relatively high mean calcification rates of 1.17 g cm^−2^ year^−1^ (Perry et al., 2012). Variation in *Orbicella* calcification rates has previously been associated with variability in environmental factors such as temperature and thermal stress, nutrient levels, aragonite saturation state, and light availability (Carricart‐Ganivet, Cabanillas‐Terán, Cruz‐Ortega, & Blanchon, 2012; Carricart‐Ganivet & Merino, 2001; Dunn, Sammarco, & LaFleur, 2012; Manzello, Enochs, Kolodziej, & Carlton, 2015). For uncaged colonies, we observed *O. faveolata* calcification rates (mean 1.35 ± 0.11 g cm^−2^ year^−1^) similar to the Caribbean average, while in both full and half cages these were reduced by 43% to 0.77 ± 0.19 g cm^−2^ year^−1^ (Figure 2d). These findings imply that coral calcification is strongly mediated by macroalgal overgrowth (Figure 2) compared with other factors. For example, Carricart‐Ganivet et al. (2012) predict that sea surface temperature increase will not have a similar impact on *Orbicella* spp. calcification rates until the year 2100. Recently, Cramer, O'Dea, Clark, Zhao, and Norris (2017) reported a positive causal effect of parrotfishes on reef accretion at millennial timescales. Linking our findings with processes occurring at geological scales, and investigating the interaction of herbivore loss with global climate change and other anthropogenic stressors should be a priority in order to conserve community calcification in the mid and long term (Kuffner & Toth, 2016).

Ecosystem processes are often scale‐dependent (Hewitt, Thrush, & Lundquist, 2010; Levin, 1992). External drivers and internal feedbacks affecting coral‐algal dynamics in reef systems vary over both temporal and spatial scales (Adam, Burkepile, Ruttenberg, & Paddack, 2015). Our finding that fish herbivory has a significant impact on algal proliferation is consistent with previous small‐scale exclusion experiments (Figure 1; Burkepile & Hay, 2006), but contrasts with large‐scale correlational studies that show no evidence of herbivore control of algae on Caribbean reefs (Cox et al., 2017; Russ et al., 2015; Suchley et al., 2016). On small spatial and temporal scales, herbivorous fishes may control algal growth while at larger and longer scales nutrient input, herbivore depletion below critical thresholds and other ecological processes may promote algal proliferation (Mumby, Hastings, & Edwards, 2007; Paddack et al., 2009).

In this study, in situ herbivore feeding observations suggest that turf grazing was primarily performed by the surgeonfish *Acanthurus bahianus* and juvenile *Scarus iseri* parrotfishes. *Acanthurus* spp. have a mixed diet (Adam, Burkepile, et al., 2015; Burkepile & Hay, 2011) and may also have been responsible for (unobservable) macroalgal propagule removal from uncaged control plates; however, longer observation periods would be required to detect this effect. *Scarus* parrotfishes are primarily turf grazers (Adam, Kelley, et al., 2015; Burkepile & Hay, 2010) and small‐bodied *S. iseri* have been shown to exert substantial grazing pressure on certain Caribbean reefs (Kuempel & Altieri, 2017). Herbivore exclusion encouraged growth of *Dictyota* spp., brown fleshy macroalgae common to Caribbean reefs (Littler, Littler, & Brooks, 2006; Quan‐Young, Diaz‐Martin, & Espinoza‐Avalos, 2004; Renken, Mumby, Matsikis, & Edwards, 2010). *Dictyota* spp. domination may result from their ability to grow and colonize substrata faster than many other benthic components as their branching form facilitates overgrowth and fragmentation enables rapid dispersal (Beach et al., 2003; Ferrari et al., 2012; Herren, Walters, & Beach, 2006). These results agree with previous studies that suggest a mix of herbivorous fishes are required in order to both prevent algal colonization, graze turf algae, and crop existing macroalgal stands (Burkepile & Hay, 2008; Rasher, Hoey, & Hay, 2013). This has important implications for coral communities as turf grazing liberates substrate space for coral recruits, and cropping of macroalgal stands reduces competition with adult coral colonies enhancing coral fecundity, growth, and survival (Box & Mumby, 2007; Kuffner et al., 2006; McCook et al., 2001).

Increased macroalgal (specifically *Dictyota*) overgrowth in herbivore exclusion cages suppressed *Orbicella faveolata* growth rates (Figures 2 and 3; Table 3). However, it is unclear which competitive mechanism—smothering, shading, abrasion, allelopathic, or enhanced microbial activity—is responsible for diminished coral growth and tissue mortality (Chadwick & Morrow, 2011; McCook et al., 2001). Recent studies suggest that abrasion may have a limited impact as a mechanism for coral‐algal interaction (Diaz‐Pulido, Harii, McCook, & Hoegh‐Guldberg, 2010; Rasher & Hay, 2010). We observed that coral growth was unrelated to overall plate algal cover, and consequently, direct contact may be necessary for other coral‐algal interaction mechanisms such as allelopathy, microbial transmission, and local hypoxia to take effect (Barott et al., 2012; Nugues, Smith, van Hooidonk, Seabra, & Bak, 2004; Rasher & Hay, 2010; Wolf, Wild, & Nugues, 2012). The negative effects of turf algal overgrowth were negligible in this study potentially due to the low severity of interactions or the inconsistent nature of hypoxia generated at the coral‐algal interface (Wangpraseurt et al., 2012).

While some herbivore exclusion studies have found evidence of coral growth suppression, other studies report no impact of reduced herbivory levels on coral growth. This is likely a result of the specific nature of the interaction between corals and macroalgae that proliferate as a result of reduced herbivory (McCook et al., 2001). Coral‐algal interaction outcomes depend upon coral growth form (branching, massive, encrusting, digitate), condition and life stage; algal morpho‐functional group (foliose, filamentous, calcareous, crustose); species identity; and environmental factors such as nutrient and light levels (Fong & Paul, 2011; McCook et al., 2001). For example, canopy‐forming algae (e.g., *Sargassum* spp.), foliose macroalgae (e.g., *Dictyota* spp. or *Lobophora* spp.), or filamentous turf algae, generate distinct physical coral interactions due to their varying morphologies (Box & Mumby, 2007; River & Edmunds, 2001; Titlyanov, Yakovleva, & Titlyanova, 2007). Exclusion studies typically employ small, short‐lived, “weedy” species such as Caribbean *Porites* spp. (Burkepile & Hay, 2010; McClanahan et al., 2003; Miller & Hay, 1998; Sotka & Hay, 2009) which may be more susceptible to algal overgrowth and contribute little to reef framework (Alvarez‐Filip et al., 2013; Tanner, 1995). In one of the few studies of important reef‐building corals, Lirman (2001) explored the impact of herbivore exclusion on large (>1 m diameter) *O. faveolata* colony perimeters. In the absence of herbivores, Lirman (2001) observed increases in filamentous algae, corticated red macroalgae, and *Dictyota* along coral perimeters; resulting in greater live tissue margin retreat and mortality rates. For *O. faveolata* fragments, our findings contrast Vu et al. (2009) who report no significant impact of macroalgae on coral growth. However, this may be because calcification rates were determined after only 21 days in that study.

Herbivore exclusion has become a standard technique to experimentally assess herbivores’ ability to graze reef algae. However, by definition, such studies involve manipulation and consequently are susceptible to confounding factors (McCook et al., 2001). Accordingly, these studies often include procedural controls in an attempt to control for experimental manipulation. In this study, we adopted the common procedural control of half exclusion cages to allow fish access from above, but retain the manipulation and barrier and shading effects of side panels. However, we observed that half cage procedural controls allowed an intermediate level of herbivory (Figure 1d), similarly to other studies (Castro‐Sanguino, Lovelock, & Mumby, 2016; Diaz‐Pulido & McCook, 2003; Ferrari et al., 2012). The effectiveness of the procedural control varied with algal type. Turf algal cover was significantly higher in full cages than half cages (Figure 1f) whereas *Dictyota* macroalgae grew in both full exclusion cages and half cage procedural controls (Figure 1e). Supported by in situ observations, it is likely that larger macroalgae‐browsing *Sparisoma* parrotfishes were either unable or unwilling to enter half cages (Diaz‐Pulido & McCook, 2003). Consequently, similar levels of macroalgal‐coral overgrowth and coral growth rates were observed in full and half cages (Figure 2c,d; Table 3), suggesting that half cages may have limited efficacy as procedural controls. Two further options for procedural controls were cages with top panels only to test the effect of side panels and plates with steel reinforcing bars only to test for any effect of the bars themselves; however, insufficient coral colonies were available for these.

Few studies have evaluated the effect of caging on the enclosed environment. Water movement has been assessed using different techniques and found not to be significantly affected within exclusion cages (Castro‐Sanguino et al., 2016; Lewis, 1986; Vermeij et al., 2010). Studies report differing effects of caging on incident light levels, potentially due to cage construction, ambient light levels, other environmental conditions, or measurement procedure (Castro‐Sanguino et al., 2016; Ferrari et al., 2012; Vermeij et al., 2010). In this study, we used Hobo data loggers to observe that mean light intensity incident at uncaged control plates on a particular day was slightly (4.7%) higher than within half cages, which in turn was (10.9%) greater than within full cages. However, coral calcification rates are relatively insensitive to such minor reductions in light intensity and consequently cage effects could only account for a small fraction of the differences in calcification between herbivore treatments (Venti, Andersson, & Langdon, 2014).

Nutrient enrichment of coastal waters and overfishing have promoted macroalgal proliferation on Caribbean reefs (Jackson et al., 2014; Lapointe, Barile, Littler, & Littler, 2005; Lapointe et al., 2010). Macroalgae compete with corals, reducing fitness and suppressing growth (Chadwick & Morrow, 2011; Fong & Paul, 2011; McCook et al., 2001). In this study, we have shown that fish herbivory combats macroalgal growth and facilitates *Orbicella* coral calcification, a major contributor to Caribbean reef framework. However, in recent decades, Caribbean reefs have lost both overall coral cover and suffered shifts to more short‐lived coral species which contribute less to reef framework. Consequently, herbivorous fishes and macroalgal control are increasingly important for today's Caribbean reefs. Policy makers and local managers should consider measures to protect herbivorous fishes and reduce macroalgal proliferation in order for reefs to continue to grow, function, and survive anthropogenic sea‐level rise. Fisheries management strategies including quotas, size limits, and gear restrictions can serve to protect fish populations. Spatial management such as the implementation of marine protected areas, and no‐take zones are also useful tools if well enforced. However, these measures must be combined with watershed management strategies to address uncontrolled coastal development and inadequate wastewater treatment which have caused large‐scale nutrient enrichment of coastal Caribbean waters (Risk, 2014).

## AUTHOR CONTRIBUTIONS

A.S. and L.A.‐F. conceived the study. A.S. performed fieldwork and analyzed the data. Both authors wrote and reviewed the manuscript.

## CONFLICT OF INTEREST

None declared.
