# Supplementary material for: Herbivory facilitates growth of a key reef‐building Caribbean coral
Source: Ecol Evol. 2017 Nov 22;7(24):11246–56. doi: 10.1002/ece3.3620 (PMC5743540; doi:10.1002/ece3.3620)
Supplement: Supplementary file 1 [file ECE3-7-11246-s001.docx]

**Herbivory facilitates growth of a key reef-building Caribbean coral**

Adam Suchley & Lorenzo Alvarez-Filip

**Supplementary Information**

**C)**

**B)**

**A)**


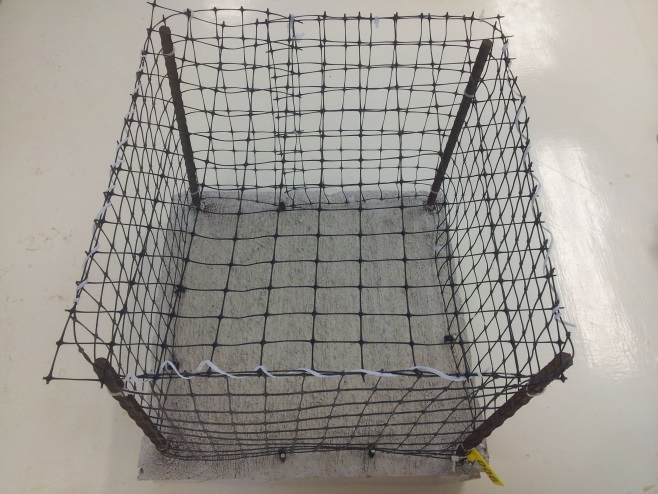

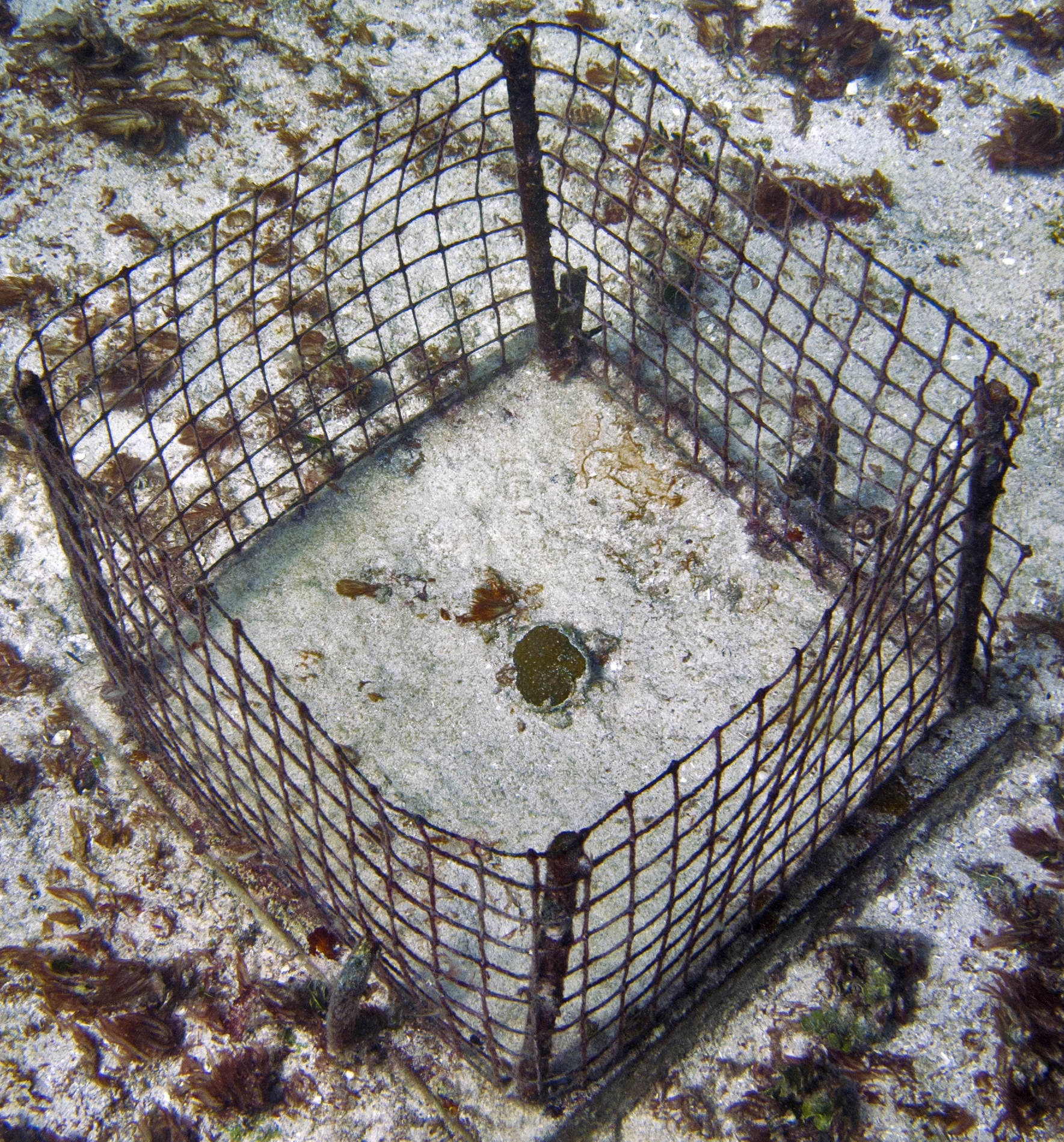

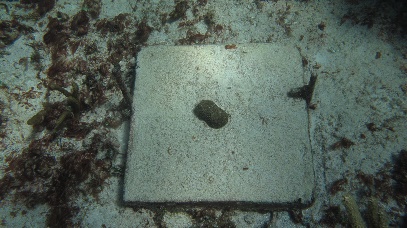


**Figure S1. Herbivorous fish exclusion cages.** (A) A full exclusion cage. Cages were built upon 36 cm-square concrete plates, with 30 cm vertical rebars and 3 cm x 2 cm polyethylene mesh. The top panel was attached to cage sides using twist ties to allow access for mesh cleaning and plate monitoring. Small (approximately 5 cm maximum diameter) *O. faveolata* coral colonies were affixed to plate centres using underwater epoxy plasticine. (B) A half exclusion cage *in situ* with an *Orbicella faveolata* coral colony. Half cages acted as procedural controls and were identical to full cages without a top panel to allow fish access. (C) An uncaged control plate *in situ* with an *Orbicella faveolata* coral colony. Controls were merely concrete plates without any caging or rebars.

**Figure S2. Mean plate short and long turf algal cover.** Mean (± s.e.m.) short (< 0.5 cm) and long (≥ 0.5 cm) algal turf cover over the observation period for full exclusion cages (n = 6), half cages (n = 6) and uncaged control plates (n = 6).

**Figure S3. Herbivory pressure.** Mean (± s.e.m.) herbivory pressure for full exclusion cages (n = 3), half cages (n = 3) and uncaged control plates (n = 3) based on *in situ* observations. Herbivory pressure expressed as bite rate per hour per plate standardised (multiplied) by grazing herbivorous fish biomass.

**Figure S4. Initial *Orbicella faveolata* colony size.** (A) Mean (± s.e.m.) initial coral colony mass. (B) Mean (± s.e.m.) initial coral colony polyp count. (C) Mean (± s.e.m.) initial coral colony planar area. All shown for full exclusion cages (n = 6), half cages (n = 6) and uncaged control plates (n = 5, as one colony was lost after two months and is therefore not included in the coral growth calculations).
